# The 1α,25-dihydroxyvitamin D3 modulates T cell activation and immune checkpoint pathways in human T cells

**DOI:** 10.3389/fimmu.2026.1802168

**Published:** 2026-07-09

**Authors:** Lisa Isdraele Romano, Ilenia Aversa, Antonio Abatino, Costanza Maria Cristiani, Giulio Cesare Antico, Debora Gentile, Caterina Giordano, Emilio Straface, Michael Marrano, Elvira Angotti, Camillo Palmieri, Raffaella Gallo, Giuseppe Fiume

**Affiliations:** 1 Department of Experimental and Clinical Medicine, University of Catanzaro “Magna Græcia”, Catanzaro, Italy; 2 Neurosciences Research Centre, Department of Medical and Surgical Sciences, University of Catanzaro “Magna Græcia”, Catanzaro, Italy; 3 Laboratory of Clinical Biochemistry, AOU “Renato Dulbecco” Hospital, Catanzaro, Italy

**Keywords:** 1,25(OH)2 vitamin D, CTLA-4, effector T cell activity, immune checkpoint regulation, PD-1, TIGIT, TIM3

## Abstract

**Background:**

Vitamin D is a pleiotropic steroid hormone with immunomodulatory properties. The role of the active metabolite, 1α,25-dihydroxyvitamin D_3_ (1,25(OH)_2_ vitamin D), in regulating human T-cell effector function and immune checkpoint (IC) pathways remains poorly defined.

**Methods:**

Peripheral blood mononuclear cells (PBMCs) from healthy donors were cultured for five days in presence or absence of 1,25(OH)_2_ vitamin D (10 or 100 nM). IFN-γ production was assessed by ELISpot assay. Baseline plasma 1,25(OH)_2_ vitamin D levels were measured. IC-related gene expression was evaluated by RT-qPCR. Flow cytometry was used to assess the expression of IC, degranulation, and activation markers. FluoroSpot assays were performed to quantify interleukin secretion.

**Results:**

1,25(OH)_2_ vitamin D reduced IFN-γ expression and secretion independently of baseline circulating 1,25(OH)_2_ vitamin D levels. Dose-specific modulation of IC genes was detected, with increased PDCD1 and CTLA4 mRNA expression. Flow cytometry showed selective modulation within the CD8^+^ T-cell compartment, with increased PD-1 expression at 10 nM and increased CTLA-4 expression at 100 nM, together with decreased degranulation and activation markers. No significant changes were detected for TIM-3 or TIGIT. In CD4^+^ T cells, no significant modulation of IC molecules was observed, whereas IL-22 secretion was reduced.

**Conclusions:**

These findings support an immunomodulatory effect of 1,25(OH)_2_ vitamin D on human CD8^+^ T cells, with attenuation of effector-associated responses and modulation of checkpoint-associated pathways *in vitro*. Our results suggest that vitamin D may contribute to the regulation of inflammatory T-cell responses under controlled culture conditions. The functional and therapeutic implications of these observations require further investigation.

## 1 Introduction

The active form of vitamin D, 1,25(OH)_2_ vitamin D (1α,25-dihydroxyvitamin D_3_ or calcitriol), is a steroid hormone classically involved in the regulation of calcium and bone homeostasis (1). Its biological effects are mediated by the vitamin D receptor (VDR), a ligand-activated intracellular receptor that functions as a transcription factor (2, 3). Upon ligand binding, VDR translocates to the nucleus and regulates gene expression through complex transcriptional programs that are highly dependent on cellular context, chromatin accessibility, and ligand concentration. Time-resolved analyses have shown that VDR signaling involves the rapid induction of primary target genes encoding transcriptional regulators, which subsequently control secondary gene networks, enabling context- and dose-dependent biological responses (4).

Beyond its endocrine role, vitamin D has emerged as an important modulator of immune homeostasis. VDR expression is widespread across the immune system, including T and B lymphocytes, monocytes, macrophages, and dendritic cells, highlighting the immunological relevance of vitamin D signaling (5, 6). Within the innate immune compartment, 1,25(OH)_2_ vitamin D promotes monocyte differentiation and enhances antimicrobial functions (7–9). In antigen-presenting cells (APCs), vitamin D signaling downregulates major histocompatibility complex class II molecules, co-stimulatory receptors, and pro-inflammatory cytokines such as IL-12 and IL-23, thereby promoting a more tolerogenic phenotype (10, 11).

The immunomodulatory effects of 1,25(OH)_2_ vitamin D on adaptive immunity have been most extensively studied in CD4^+^ T cells. In this context, vitamin D signaling has been shown to suppress CD4^+^ T cell proliferation and to skew their differentiation away from pro-inflammatory Th1, Th17, and Th22 lineages while favoring Th2-associated cytokine responses (12–14). These functional changes are underpinned by extensive epigenetic remodeling, including alterations in super-enhancer landscapes and the coordinated recruitment of transcription factors such as c-JUN, STAT3, and BACH2 together with VDR (15).

In contrast, the role of 1,25(OH)_2_ vitamin D in CD8^+^ T cells has been less extensively explored, despite several observations suggesting that this cell population may be particularly responsive to vitamin D signaling. For instance, both resting and activated CD8^+^ T cells express higher levels of VDR compared to CD4^+^ T cells (16), implying a potential involvement of Vitamin D signaling in CD8^+^ T cell biology. Moreover, CD8^+^ T cells from VDR-deficient mice display enhanced proliferative capacity *in vivo* and *in vitro* following CD3/CD28 stimulation, supporting a role for VDR signaling as a negative regulator of CD8^+^ T cell expansion (17). However, the downstream pathways and functional consequences of VDR activation in human CD8^+^ T cells remain largely unexplored.

CD8^+^ T cells play a central role in antiviral and antitumor responses by mediating antigen-specific cytotoxic response following T cell receptor (TCR) engagement (18–20). In cancer and chronic inflammatory settings, persistent antigen exposure drives CD8^+^ T cells toward a dysfunctional or exhausted state characterized by impaired effector cytokine production and sustained expression of inhibitory immune checkpoint (IC) receptors (21, 22). Inhibitory molecules such as programmed cell death protein 1 (PD-1), and cytotoxic T lymphocyte–associated antigen 4 (CTLA-4) attenuate TCR signaling and cytokine production, thereby limiting immunopathology while constraining protective immunity. Additional inhibitory receptors, including T-cell immunoglobulin and mucin domain-containing protein 3 (TIM-3) and T cell immunoreceptor with Ig and ITIM domains (TIGIT), further fine-tune T cell responses in a context-dependent manner. Importantly, IC expression does not necessarily reflect terminal exhaustion but may instead represent graded and reversible regulatory states. IC pathways have gained particular prominence due to their therapeutic targeting in cancer (23), yet they are also involved in immune homeostasis under physiological conditions.

Despite the well-established immunomodulatory properties of vitamin D, its role in regulating IC pathways in human CD8^+^ T cells remains poorly defined (24). While vitamin D–dependent transcriptional programs have been linked to immune tolerance, direct evidence for selective modulation of inhibitory checkpoint receptors is limited, particularly in primary human T cells.

In the present study, we examined the effects of 1,25(OH)_2_ vitamin D on effector function and IC regulation in human peripheral blood T cells, with a particular focus on CD8^+^ T cells. Using peripheral blood mononuclear cells (PBMCs) from healthy donors, we combined functional assays, gene expression analyses, and surface protein profiling to assess how vitamin D signaling shapes T cell effector and inhibitory programs.

## 2 Materials and methods

### 2.1 Study design and ethics

The study was approved by the Regional Ethical Review board of Regione Calabria (protocol n.69/2023). The research was carried out according to the principles of the Declaration of Helsinki, as revised in 2013. Informed consent was obtained for all the participants in the study.

### 2.2 1,25(OH)_2_ vitamin D measurement

Measurements of plasma 1,25(OH)_2_ vitamin D were performed as previously described (25), using a chemiluminescent immunoassay (CLIA) on the DiaSorin Liaison^®^ XL analyzer (DiaSorin S.p.A., Saluggia (VC), Italy). The DiaSorin Liaison^®^ XL 1,25 Dihydroxyvitamin D assay is a modified 3-step sandwich assay employing a recombinant fusion protein to capture the 1,25(OH)_2_ vitamin D molecule and a mouse monoclonal antibody that specifically recognizes the resulting complex. The manufacturer declares a measurement range of 5.0–200 pg/mL and the limit of quantification (LOQ) of 5.0 pg/mL. All the measurements are reported in **Supplementary Table 1**.

### 2.3 Peripheral blood mononuclear cells isolation

Peripheral blood mononuclear cells (PBMCs) were obtained from buffy coat preparations derived from peripheral blood collected in CPDA-1-anticoagulant blood bags from healthy donors. PBMCs were isolated by density gradient using Histopaque-1077 (code 10771; Sigma-Aldrich, Darmstadt, Germany), according to the manufacturer’s instructions and as previously reported (26, 27). Isolated PBMCs were cultured in RPMI 1640 medium supplemented with 10% fetal bovine serum (FBS), 2 mM L-glutamine, and 100 U/mL penicillin/streptomycin. To support cell viability, recombinant human IL-2 (30 U/mL; #200-02, PeproTech) was added to the culture medium every 48 h, as previously described (19). Cells were maintained at 37 °C in a humidified atmosphere containing 5% CO_2_.

### 2.4 PBMCs treatment with 1,25(OH)_2_ vitamin D

Freshly isolated PBMCs were plated in 12-well plates (2x10^6^ PBMCs/well) in complete RPMI medium and treated once on day 0 with 1,25(OH)_2_ vitamin D (#sc-202877B, Santa Cruz Biotechnology) at final concentrations of 10nM or 100nM, while untreated cells cultured under the same conditions were used as control (CTR). Cells were collected on day 5 for IFN-γ ELISpot assay, flow cytometric analysis and RNA extraction.

### 2.5 Enzyme-linked immunoSpot assay

Enzyme-Linked ImmunoSpot (ELISpot) path kit (Cat. No. 3420-2AST-10, Mabtech, Sweden) was used for the detection of interferon gamma (IFN-γ)-secreting cells in response to stimulation, according to manufacturer’s instructions. Briefly, 1x10^5^ cells/well were seeded in 96-well plates pre-coated with human IFN-γ antibody (mAb 1-D1K) and incubated in a 37°C humidified incubator with 5% CO2 in the presence of appropriate stimuli. A peptide pool containing 42 peptides from Cytomegalovirus (CMV) (PepPool: CMV (CD4 and CD8), human, #36-19-1, Mabtech, Sweden) was used, according to the manufacturer’s specifications. After 18 hours, the cells were removed and the plates washed 5 times. Plates were incubated with a detection antibody (7-B6-1-biotin) for 2 hours at room temperature, followed by a 1-hour incubation at room temperature with the streptavidin-ALP. Finally, a filtered substrate solution (BCIP/NBT-plus) was added to each well until spots were visible. Spots corresponding to stimulated cells secreting IFN-γ were counted using the IRIS 2 FluoroSpot/ELISpot reader (Mabtech, Sweden). The IFN-γ-ELISPot data were reported as Spot Forming Units × 10^6^ PBMCs (SFU/10^6^).

### 2.6 RNA extraction and RT-qPCR

Total RNA was isolated from PBMC samples using TRIzol™ reagent (#15596018, Invitrogen, ThermoFisher Scientific, Milan, Italy) as previously described (28) and reverse-transcribed into complementary DNA (cDNA) using the High-Capacity cDNA Reverse Transcription Kit (ThermoFisher Scientific, Milan, Italy), according to the manufacturer’s instructions. The mRNA expression levels of genes of interest were analyzed by quantitative real-time PCR (RT-qPCR) using yourSIAL^®^ Green Mix 2× (SIAL srl) with ROX as passive reference dye. Primer sequences are reported in **Supplementary Table 2**. Gene expression was normalized to the endogenous control β-actin, and relative mRNA expression levels were calculated using the 2^-^ΔΔCT method. Quantitative PCR analyses were performed using the Applied Biosystems QuantStudio 3 Real-Time PCR System.

### 2.7 Flow cytometry analysis

After 5 days of culture, 4×10^5^ PBMCs were collected, washed in phosphate-buffered saline containing 2% fetal bovine serum (FACS buffer), and stained with Fixable Viability Stain 780 (1:1000, Cat. No. 565388, BD Horizon) for 15 min in the dark, prior to surface antibody staining. Cells were washed twice with FACS buffer and incubated with antibody cocktails for 30 min at 4 °C in the dark. Antibody titrations were performed before experiments to determine optimal staining concentrations. After staining, cells were washed, resuspended in 300 μL FACS buffer, and acquired on a BD FACS LSR Fortessa X-20 (BD Biosciences, San Diego, CA). Antibodies used for flow cytometry analyses are listed in **Supplementary Table 3**.

### 2.8 CD107a degranulation assay

After 5 days of culture, 1×10^6^ PBMCs were stimulated with human T-activator CD3/CD28 Dynabeads (ThermoFisher Scientific, Cat. No. 11132D) for 4 h according to the manufacturer’s instructions, while parallel unstimulated samples served as negative controls. Cells were then washed with FACS buffer and stained sequentially with anti-CD107a antibody (Invitrogen, Cat. No. 14-1079-80), AlexaFluor-647 secondary antibody (Abcam, Cat. No. ab150115), and anti-CD3 and anti-CD8 antibodies (30 min each at 4 °C). Finally, cells were resuspended in 300 μL FACS buffer and acquired as described above.

### 2.9 FluoroSpot Plus assay

FluoroSpot assays were performed using FluoroSpot Plus kits (Mabtech, Sweden) as detailed in the **Supplementary Materials and Methods**.

### 2.10 Statistical analysis

Statistical analyses were performed using GraphPad Prism 8 and R software (version 4.2.3) (29). Detailed information regarding statistical analyses, normalization procedures, and multiple-comparisons testing is provided in the **Supplementary Materials and Methods**.

## 3 Results

### 3.1 Effect of 1,25(OH)_2_ vitamin D on IFN-γ production and secretion by PBMCs

**Figure 1:**
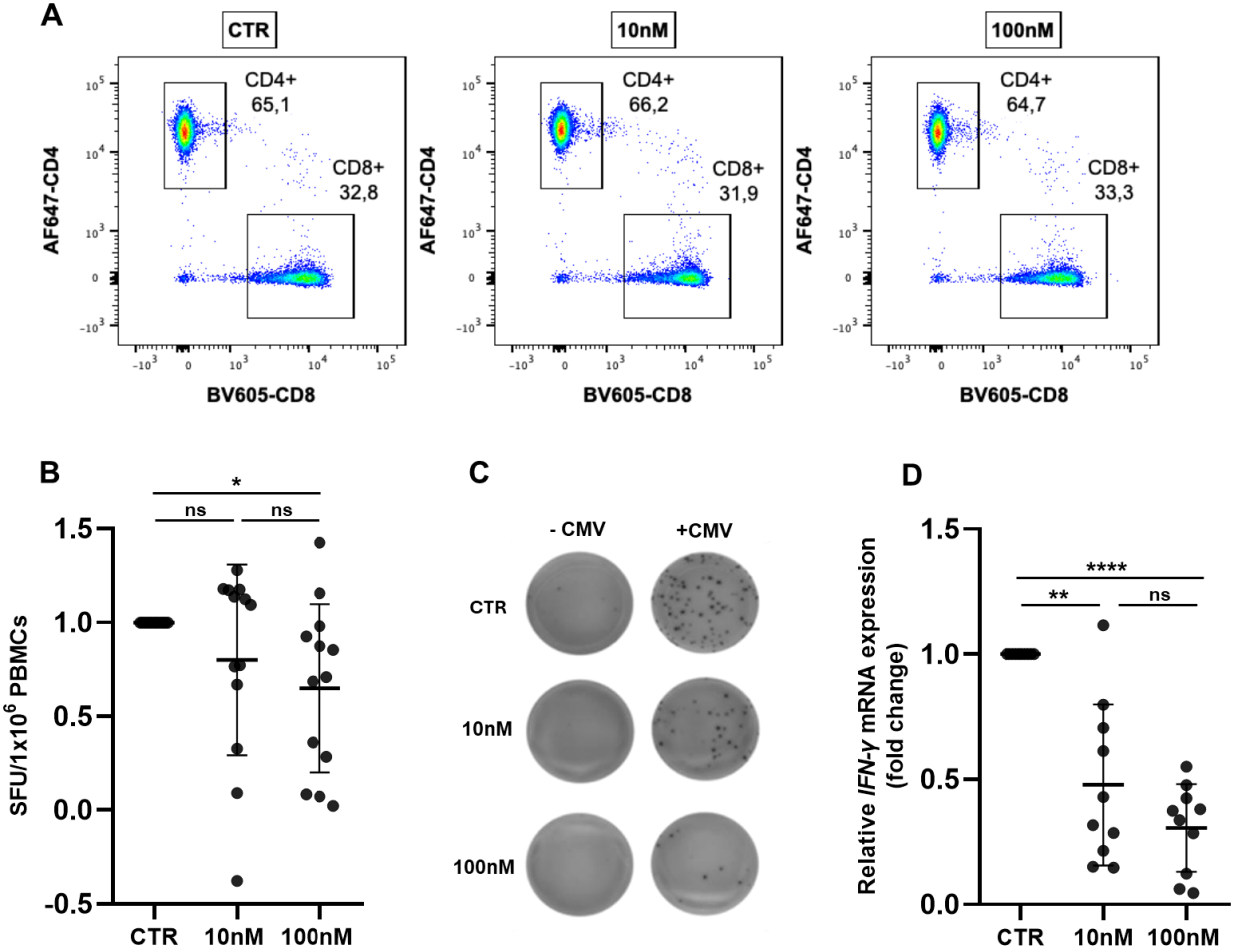
1,25(OH)_2_ vitamin D modulates IFN-γ responses in PBMCs. **(A)** Representative flow cytometry images showing the percentages of CD4^+^ and CD8^+^ cells within the T lymphocyte population gated as CD3^+^. **(B)** Quantification of IFN-γ secretion by PBMCs from 13 donors measured by IFN-γ ELISpot assay after stimulation in the absence (CTR) or presence of 1,25(OH)_2_ vitamin D (10 or 100 nM). Data were normalized to the untreated control (CTR = 1) and are shown as individual donors with mean ± SD. SFU, Spot Forming Units. **(C)** Representative IFN-γ ELISpot results from one donor in the absence (-CMV) or presence (+CMV) of CMV peptide pool, under untreated control (CTR) or following treatment with 1,25(OH)_2_ vitamin D (10 or 100 nM). **(D)** IFN-γ mRNA expression measured by qPCR in PBMCs from 10 of the same donors and expressed as fold change relative to CTR. Individual donor values are shown with mean ± SD. Statistical analysis was performed using a repeated-measures one-way ANOVA with Geisser–Greenhouse correction followed by Tukey’s *post hoc* test. * p-value <0.05, ** p-value <0.01, **** p-value <0.001.

To assess the long-term effect of 1,25(OH)_2_ vitamin D on T cell activation, PBMCs from 13 healthy donors were isolated and cultured for 5 days in the absence (CTR) or presence of 1,25(OH)_2_ vitamin D (10 or 100 nM). These concentrations fall within the widely used 10–100 nM range reported to induce VDR activation and transcriptional responses in primary human PBMC and T cell cultures (5, 13, 30, 31). Two concentrations were included to explore potential dose-dependent responses. The typical distribution of CD4^+^ and CD8^+^ cells within the CD3^+^ lymphocyte population was observed in our samples (**Figure 1A**). T cell activation was evaluated by measuring IFN-γ secretion in an ELISpot assay that predominantly reflects T-cell activity within PBMC cultures. Cytomegalovirus (CMV) peptide pools with broad HLA coverage were used as a positive control due to the high prevalence of CMV infection within the population, and the resulting high frequency of CMV-specific IFN-γ-producing memory T cells in human PBMCs (32). Treatment with 100 nM 1,25(OH)_2_ vitamin D significantly reduced IFN-γ secretion compared with untreated controls, resulting in an approximately 35% reduction in IFN-γ SFU counts (mean difference: 0.3510, 95% CI: 0.01965–0.6823; p = 0.0378). A more modest reduction (~20%) was also observed following treatment with 10 nM 1,25(OH)_2_ vitamin D, although this did not reach statistical significance (mean difference: 0.1991, 95% CI: −0.1764 to 0.5747; p = 0.3646) (**Figures 1B, C**). Absolute IFN-γ ELISpot values for individual donors are reported in **Supplementary Table 4**.

To further investigate whether 1,25(OH)_2_ vitamin D affected IFN-γ expression at the transcriptional level, we evaluated IFN-γ mRNA expression by qPCR. Due to limitations in RNA quantity and/or quality, this analysis could be performed on only 10 of the 13 donors included in the study (**Supplementary Table 5**). As shown in **Figures 1D**, IFN-γ mRNA levels were significantly reduced at both 10 nM and 100 nM compared to CTR. In particular, IFN-γ expression decreased by approximately 52% at 10 nM (mean difference: 0.5223, 95% CI: 0.2385–0.8062; p = 0.0016) and by 69% at 100 nM (mean difference: 0.6943, 95% CI: 0.5398–0.8488; p < 0.0001).

### 3.2 Association between baseline vitamin D levels and IFN-γ response to 1,25(OH)_2_ vitamin D

To evaluate whether the magnitude of the *in vitro* response to 1,25(OH)_2_ vitamin D was influenced by baseline vitamin D levels, circulating plasma 1,25(OH)_2_ vitamin D levels were measured in 10 donors from the same cohort (**Supplementary Table 1**).

**Figure 2:**
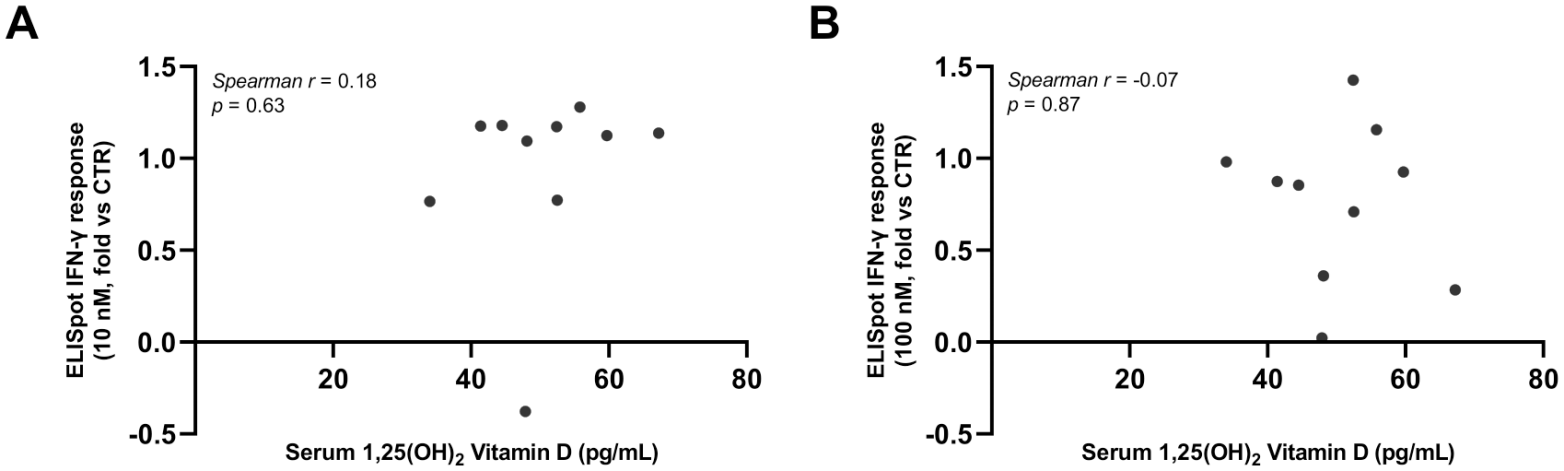
Baseline serum 1,25(OH)_2_ vitamin D levels do not predict IFN-γ responsiveness to vitamin D *in vitro*. Correlation between baseline serum 1,25(OH)_2_ vitamin D levels and IFN-γ production by PBMCs following *in vitro* treatment with 1,25(OH)_2_ vitamin D Scatter plots show the relationship between serum 1,25(OH)_2_ vitamin D concentrations and IFN-γ ELISpot responses (expressed as fold change vs CTR) after stimulation with 10 nM **(A)** or 100 nM **(B)** 1,25(OH)_2_ vitamin D Each dot represents an individual donor (n = 10). Correlations were assessed using Spearman’s rank correlation.

Correlation analyses were then performed between plasma 1,25(OH)_2_ vitamin D levels and IFN-γ production measured by ELISpot following *in vitro* stimulation with either 10 nM or 100 nM 1,25(OH)_2_ vitamin D. As shown in **Figure 2**, no significant correlations were observed at either concentration (Spearman r = 0.18, p = 0.63 for 10 nM; r = −0.07, p = 0.87 for 100 nM; n=10). These results indicate that IFN-γ responses in our experimental system were independent of baseline vitamin D status and primarily driven by *in vitro* stimulation.

### 3.3 Effect of 1,25(OH)_2_ vitamin D on immune checkpoint gene expression

**Figure 3:**
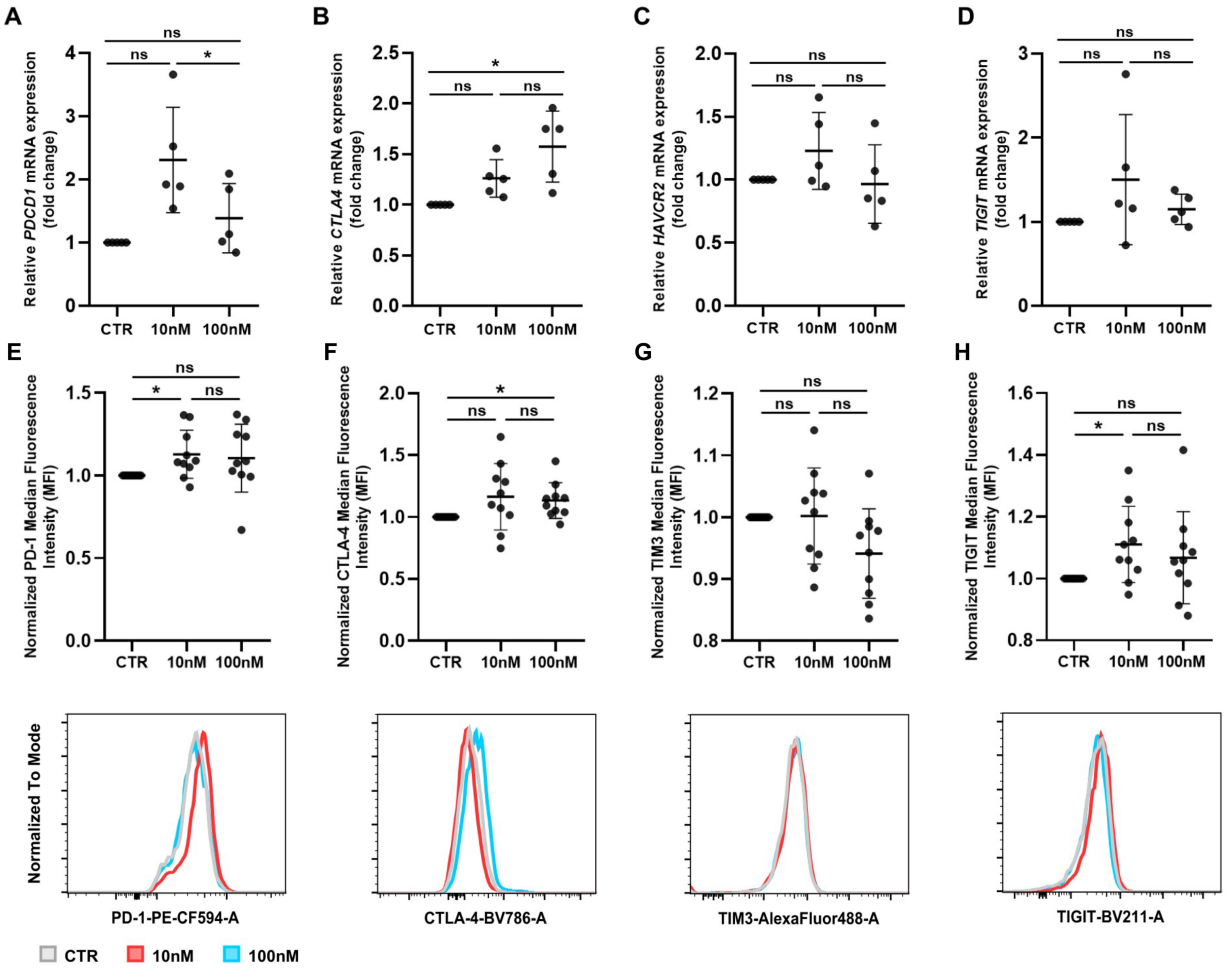
1,25(OH)_2_ vitamin D modulates immune checkpoint gene expression in PBMCs and receptor expression on CD8^+^ T cells. Relative mRNA expression of *PDCD1* **(A)** *CTLA4* **(B)** *HAVCR2* **(C)** and *TIGIT* **(D)** in PBMCs (n=5) stimulated in the absence (CTR) or presence of 1,25(OH)_2_ vitamin D (10 or 100 nM) was measured by qPCR (upper panels). Data are expressed as fold change relative to control (CTR = 1). In the middle panels, the surface expression of PD-1 **(E)**, CTLA-4 **(F)**, TIM-3 **(G)**, and TIGIT **(H)** on CD8^+^ T cells (n=10) was assessed by flow cytometry after PBMCs stimulation in the absence (CTR) or presence of 1,25(OH)_2_ vitamin D (10 or 100 nM). Data are shown as normalized median fluorescence intensity (MFI) values relative to untreated controls (CTR = 1). Lower panels show representative histograms for each analyzed IC marker. Each dot represents an individual donor, and data are expressed as mean ± SD. Statistical analysis was performed using repeated-measures one-way ANOVA with Geisser–Greenhouse correction, followed by Tukey’s *post hoc* test. *p-value <0.05.

To explore potential molecular mechanisms underlying the reduced T cell effector response, the expression of selected IC genes was analyzed by qPCR in PBMCs from 5 donors belonging to the same donor cohort (**Supplementary Table 5**). This sub-cohort was selected solely by sample availability and technical constraints, and missing data occurred due to limited RNA yield. Analysis of *PDCD1* mRNA expression revealed a trend toward increased expression following treatment with 10 nM 1,25(OH)_2_ vitamin D (2.3-fold vs CTR), whereas a more modest increase was observed at 100 nM (1.4-fold vs CTR). Although neither treatment significantly differed from CTR, *PDCD1* expression was significantly higher in PBMCs treated with 10 nM compared with 100 nM 1,25(OH)_2_ vitamin D (mean difference: 0.9196, 95% CI: 0.2204–1.619; p = 0.0204), as shown in **Figure 3A**.

CTLA4 mRNA expression was increased in PBMCs treated with 1,25(OH)_2_ vitamin D, with higher expression levels observed at 100 nM (1.6-fold vs CTR) compared with 10 nM (1.3-fold vs CTR). The increase observed at 100 nM was statistically significant (mean difference: −0.5754, 95% CI: −1.134 to −0.01657; p = 0.0456), whereas the increase observed at 10 nM did not reach statistical significance (**Figure 3B**).

In contrast*, HAVCR2* and *TIGIT* mRNA levels showed only minor variations across treatment conditions, and no statistically significant differences were observed following treatment with 1,25(OH)_2_ vitamin D at either 10 nM or 100 nM compared with untreated controls (**Figures 3C, D**).

### 3.4 Effect of 1,25(OH)_2_ vitamin D on immune checkpoint protein expression

To investigate whether the gene expression changes detected via qPCR were reflected on the surface levels of the IC molecules PD-1, CTLA-4, TIM-3, and TIGIT on T lymphocytes we performed flow cytometry analysis. For this experiment, PBMCs were obtained from 10 donors belonging to the same donor cohort and cultured in the absence (CTR) or presence of 1,25(OH)_2_ vitamin D at 10 nM or 100 nM for 5 days. IC expression was subsequently analyzed within the CD4^+^ and CD8^+^ T-cell subsets as defined by gating strategy shown in **Supplementary Figure 1**.

As shown in **Figures 3E**, PD-1 normalized median fluorescence intensity (MFI) on CD8^+^ T cells was modestly but significantly increased following treatment with 10 nM 1,25(OH)_2_ vitamin D (1.13-fold vs CTR; mean difference: −0.1287, 95% CI: −0.2573 to −3.966 × 10^-5^; p = 0.0499). No significant differences were observed between CTR and 100 nM.

In line with the gene expression analysis, CTLA-4 normalized MFI was also increased in CD8^+^ T cells treated with 100 nM 1,25(OH)_2_ vitamin D (1.13-fold vs CTR). This increase was statistically significant compared with untreated controls (mean difference: −0.1334, 95% CI: −0.2599 to −0.006784; p = 0.0396), whereas no significant differences were observed between CTR and 10 nM or between the two vitamin D concentrations (**Figure 3F**).

In contrast, TIM-3 normalized MFI showed only minimal variations across treatment conditions (**Figure 3G**). TIGIT normalized MFI on CD8^+^ T cells was modestly increased following treatment with 10 nM 1,25(OH)_2_ vitamin D (1.11-fold vs CTR). This increase was statistically significant compared with untreated controls (mean difference: −0.1101, 95% CI: −0.2190 to −0.001132; p = 0.0478), whereas no significant differences were observed between CTR and 100 nM or between the two vitamin D concentrations (**Figure 3H**).

Importantly, 10 or 100 nM 1,25(OH)_2_ vitamin D stimulation did not induce any changes in surface levels of all the measured IC molecules in the CD4^+^ T cell population (**Supplementary Figure 2**), suggesting that prolonged exposure to 1,25(OH)_2_ vitamin D influences the expression of IC molecules only in CD8^+^ T cells.

### 3.5 Effect of 1,25(OH)_2_ vitamin D on CD4^+^ T cell-mediated cytokine production

To investigate whether the 1,25(OH)_2_ vitamin D treatment affected CD4^+^ T cell cytokine production we performed FluoroSpot Plus Assay, that allowed the parallel profiling of multiple cytokines upon TCR stimulation, including IL-4, IL-5, IL-22, IL-10, and IL-17A. For this and the following analyses, samples were obtained from a second cohort composed of 8 healthy donors (**Supplementary Table 6**), and PBMCs were exposed to only 100 nM 1,25(OH)_2_ vitamin D, to capture the greatest magnitude of cytokine modulation. Following treatment, IL-4, IL-5, and IL-17A showed a trend toward reduction compared with untreated controls, although these differences did not reach statistical significance (**Supplementary Figure 3**; **Supplementary Table 7**). Similarly, IL-10 production showed a not-significant increasing trend, likely due to high inter-donor variability. In contrast, we observed a marked and significant reduction in IL-22 secretion (~59.5% reduction vs CTR; mean difference: -0.595, 95% CI: -0.954 to -0.235; p = 0.0156). These results suggest that 1,25(OH)_2_ vitamin D influences cytokine secretion profiles associated with activated CD4^+^ T cell responses, with a marked inhibitory effect on IL-22 secretion.

### 3.6 Effect of 1,25(OH)_2_ vitamin D on CD8^+^ T cell activation and phenotype

**Figure 4:**
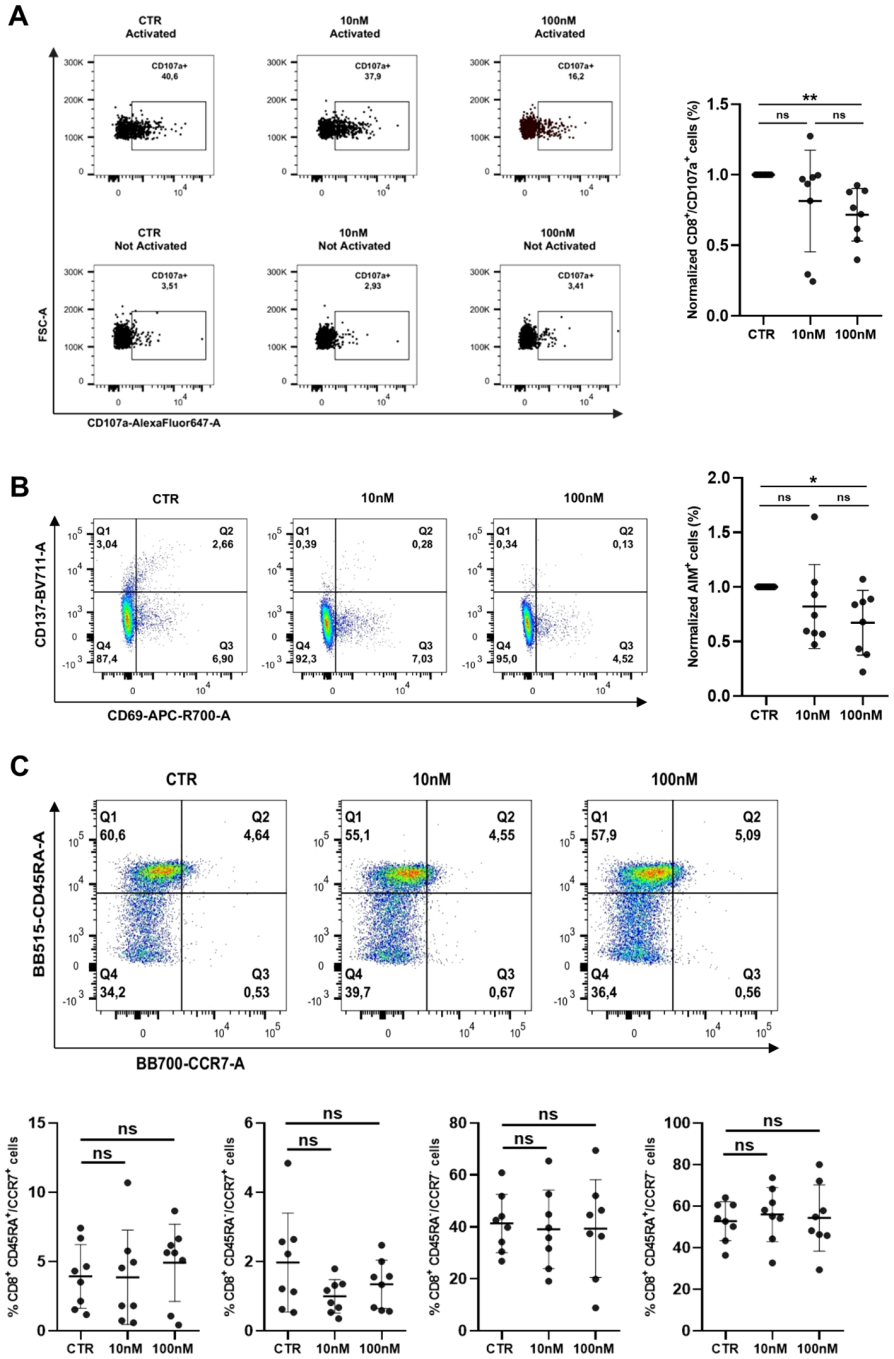
Effect of 1,25(OH)_2_ vitamin D in CD8^+^ T cell activation and phenotype. **(A)** Activation-induced degranulation, measured by CD107a membrane accumulation upon CD3/CD28 Dynabeads stimulation, was reduced in CD8^+^ T cells treated with 1,25(OH)_2_ vitamin D Representative flow cytometry plots for untreated controls (CTR), 10 nM, and 100 nM 1,25(OH)_2_ vitamin D-treated activated and not-activated CD8^+^ T cells are shown on the left, while quantification is shown on the right panel. Data are presented as normalized mean ± SD, and individual donors are shown. **(B)** The frequency of activation-induced marker positive (AIM^+^) CD8^+^ T cells was reduced following 1,25(OH)_2_ vitamin D treatment. AIM^+^ cells were defined as CD8^+^ T cells expressing at least one activation marker (CD69 and/or CD137). Representative flow cytometry dot plots are shown on the left and quantification on the right. Data are presented as normalized mean ± SD, and individual donors are shown. **(C)** The distribution of naïve, central memory, effector memory, and terminal effector memory CD8^+^ T-cell subsets was not significantly altered by 1,25(OH)_2_ vitamin D treatment. Representative flow cytometry plots for untreated controls (CTR), 10 nM, and 100 nM 1,25(OH)_2_ vitamin D treated samples are shown in the upper panels, while quantification is shown in the lower panels. Data are presented as mean percentage ± SD, and individual data points are reported. Statistical analysis was performed using repeated-measures one-way ANOVA with Geisser–Greenhouse correction, followed by Tukey’s *post hoc* test for panels **(A, B)** or Dunnett’s *post hoc* test for panel **(C)**. * p-value <0.05, ** p-value <0.01.

To further investigate the effect of the 1,25(OH)_2_ vitamin D treatment on CD8^+^ T cells we evaluated the expression pattern of CD107a, a transmembrane protein that accumulates on the plasma membrane following activation-induced degranulation (33, 34). We observed a reduction in the percentage of CD107a-positive CD8^+^ T cells upon treatment, with a more pronounced reduction observed following treatment with 100 nM 1,25(OH)_2_ vitamin D (mean difference: 0.2833, 95% CI: 0.0892–0.4773; p = 0.0087) (**Figure 4A**). We further evaluated the percentage of CD8^+^ T cells expressing at least one activation-induced marker (CD69 and/or CD137), and observed a significant reduction upon 100 nM treatment corresponding to an approximately 32.8% decrease compared with untreated controls (mean difference: 0.3280, 95% CI: 0.0192–0.6368; adjusted p = 0.0390) (**Figure 4B**). The same donors were also subjected to flow cytometric analysis to quantify the distribution of naïve (CCR7^+^/CD45RA^+^), central memory (CCR7^+^/CD45RA^-^), effector memory (CCR7^-^/CD45RA^-^), and terminal effector memory (CCR7^-^/CD45RA^+^) CD8^+^ T subsets within control and treated populations. No statistically significant differences were detected across the analyzed subsets (**Figure 4C**; **Supplementary Table 8**).

## 4 Discussion

Vitamin D is an important modulator of immune responses. In this study, we show that the active form of vitamin D, 1,25(OH)_2_ vitamin D, modulates IFN-γ expression at the transcriptional and protein levels in human PBMCs, with a reduction in IFN-γ mRNA expression already at 10 nM, and a significant decrease in IFN-γ secretion only at 100 nM treatment. This pattern suggests that transcriptional repression of IFN-γ represents a sensitive response to vitamin D, while higher concentrations are required for a measurable effect in cytokine secretion, reflecting the presence of multiple regulatory layers (30, 35).

Previous studies have shown that low serum vitamin D is associated with altered PBMC metabolism, including reduced mitochondrial respiration and increased glycolysis, suggesting that prevailing vitamin D status can modulate the metabolic activity and basal activation states of PBMCs (36). Interestingly, the magnitude of IFN-γ decrease observed *in vitro* did not correlate with baseline circulating levels of 1,25(OH)_2_ vitamin D in the donor cohort in our experimental system, indicating that the responsiveness of T cells to 1,25(OH)_2_ vitamin D is largely independent of the systemic vitamin D status and is instead determined by *in vitro* exposure to the active metabolite (37).

Because IFN-γ production reflects both the activation state and the regulation of T cells, we investigated whether 1,25(OH)_2_ vitamin D modulates IC pathways by analyzing the expression of the IC molecules PD-1, CTLA-4, TIM-3, and TIGIT. Our preliminary findings suggest that vitamin D selectively increases PD-1 and CTLA-4 expression in a concentration-specific manner, although confirmation in larger cohorts will be required. Significant differences emerged in *PDCD1* mRNA expression between vitamin D concentrations rather than between treated and untreated conditions, suggesting that 1,25(OH)_2_ vitamin D does not act as an on/off switch for PDCD1 expression but fine-tunes its transcriptional level. This is consistent with auto-regulatory mechanisms of VDR signaling, whereby higher ligand concentrations attenuate vitamin D–dependent gene transcription (3). *CTLA-4* mRNA expression was increased at 100nM vitamin D concentration, indicating a higher dose threshold for its regulation.

Flow cytometric analysis confirmed an increase in PD-1 surface expression at 10 nM, and in CTLA-4 surface expression at 100 nM treatment in CD8^+^ T cells. In contrast, 1,25(OH)_2_ vitamin D did not induce significant changes in CD4^+^ T cells.

We did not observe statistically significant changes in the expression of TIM-3 at either the transcriptional or protein level. Similarly, TIGIT showed only a modest increase in protein exposure on the membrane, which was not backed up by the exploratory mRNA expression analysis. TIM-3 and TIGIT are co-inhibitory receptors associated with antigen-experienced or chronically stimulated T cells (38, 39). Accordingly, the lack of marked TIM-3 or TIGIT increase is consistent with the absence of persistent antigenic stimulation, suggesting preferential modulation of selected checkpoint-associated pathways under these experimental conditions.

Interestingly, previous *in vitro* studies have examined these effects in isolated, strongly activated CD4^+^ T cells, where 1,25(OH)_2_ vitamin D was shown to induce PD-1 and CTLA-4 expression, reduced proliferation and pro-inflammatory cytokine production (12, 30, 40). Consistent with previous studies, we also observed a decrease in the production of inflammatory mediators like Interleukin-22 (IL-22) that is largely attributable to CD4^+^ Helper-T cells (40). However, given that the assay was performed on total PBMC cultures, a partial contribution from other T-cell populations, including CD8^+^ and unconventional T cells, cannot be excluded. Previous observations also reported enhanced PD-1 upregulation in ex vivo–stimulated CD4^+^ CD25^+^ T cells within PBMCs following vitamin D supplementation (41). In contrast, we did not observe significant checkpoint modulation in CD4^+^ T cells. This difference is likely attributable to experimental design, as prior studies largely relied on APC-free systems, whereas our analysis was conducted in PBMC cultures, preserving cellular heterogeneity. Importantly, in our experimental settings PBMCs were cultured without exogenous TCR engagement via CD3/CD28 stimulation. Under these conditions, sustained artificial activation is minimized, and increase of PD1 and CTLA-4 expression was primarily observed within the CD8^+^ T-cell compartment. These findings suggest that CD8^+^ T cells may represent a relevant target of vitamin D–mediated immunomodulatory effects in heterogeneous PBMC cultures, although additional studies will be required to determine the functional and mechanistic significance of this checkpoint-associated modulation.

We characterized CD107a surface expression on CD8^+^ T cells following treatment with 1,25(OH)_2_ vitamin D, a widely used functional marker of degranulation and cytotoxic activity (33). We observed a reduction in CD107a surface expression, suggesting a decrease in functional activity, although a deeper characterization of additional lytic effector molecules (e.g. perforin, granzyme B) will be required in the future. We also observed a reduction in the percentage of AIM^+^ CD8^+^ T cells, in line with previous studies reporting attenuated activation in T cells (31). Because our experiments were performed in total PBMC cultures, indirect mechanisms mediated by APCs could also contribute to the observed phenotypes (42–44). However, the concordant IFN-γ suppression, IC molecules upregulation, and the reduction of CD107a and activation marker exposure on CD8^+^ T cells, suggests that 1,25(OH)_2_ vitamin D reduces CD8^+^ T cell effector function.

Despite these functional changes, we did not observe substantial alterations in the distribution of naïve, central memory, effector memory, and terminal effector CD8^+^ T-cell subsets within our samples. These populations can be further subdivided into additional functional and phenotypic states based on markers that were not included in our analysis (45). Therefore, while no differences were detected among the major CD8^+^ T-cell subsets examined, we cannot exclude the possibility that changes occurred within more refined T-cell populations, potentially resulting in differential responsiveness to the compound. Previous observational studies associated higher circulating 25(OH)D levels with reduced naïve and increased effector CD8^+^ T-cell frequencies during early aging, possibly reflecting differences in study design, donor age, vitamin D metabolite analyzed, and exposure duration (46).

Beyond its immune modulatory role, *in vitro* studies on malignant cells have demonstrated that vitamin D possesses anticancer effects by multiple mechanisms such as preventing metastasis, arresting proliferation, and inducing apoptosis (47). Several epidemiologic studies suggest inverse correlations between serum 25-hydroxyvitamin D levels and cancer development, risk for cancer recurrence, and mortality (48). Moreover, *in vivo* studies have shown that vitamin D promotes CD8^+^ T cell infiltration in murine breast cancer models (49). In cancer, persistent inflammation can contribute to the establishment of a tumor-promoting microenvironment in which tumor-infiltrating T cells are recruited but progressively develop metabolic alterations that impair their antitumor activity. Under these conditions, chronic inflammatory signaling may facilitate tumor immune escape. Given its immunomodulatory properties, vitamin D could potentially help limit prolonged inflammatory responses, thereby preserving T-cell functionality while simultaneously exerting direct effects on tumor cells and potentially influencing synapse formation. Accordingly, a study compared the effectiveness of anti–PD-1 immunotherapy in patients with melanoma in relation to serum vitamin D levels, showing that maintaining the vitamin D levels within the normal range may allow the improvement of treatment outcomes (50).

Taken together, our findings support an immunomodulatory effect of 1,25(OH)_2_ vitamin D on human CD8^+^ T cells, characterized by attenuation of selected effector-associated responses together with modulation of checkpoint-associated pathways *in vitro*. These observations suggest that vitamin D may contribute to the regulation of inflammatory T-cell responses under controlled culture conditions. The potential relevance of these findings in the context of cancer immunotherapy will require further investigation.

## 5 Limitations

This study has several limitations. The initial cohort included 13 healthy adult donors selected according to standard blood donor eligibility criteria; however, the use of anonymized buffy coats prevented access to detailed demographic or clinical information. Sample numbers varied across experiments due to availability and technical constraints (**Supplementary Table 5**), and IC gene expression analysis was performed in a limited subset of samples (n = 5), thus remaining exploratory. In addition, all experiments were conducted *in vitro* and may not fully reflect *in vivo* immune responses. Validation in larger, clinically characterized cohorts and translational models will therefore be required. Finally, findings on the effects of 1,25(OH)_2_ vitamin D on CD8^+^ T-cell activation, degranulation, differentiation, and CD4^+^ T-cell cytokine production were obtained in an independent validation cohort.

## Data Availability

The original contributions presented in the study are included in the article/**Supplementary Material**. Further inquiries can be directed to the corresponding author/s.
