# Supplementary material for: The 1α,25-dihydroxyvitamin D3 modulates T cell activation and immune checkpoint pathways in human T cells
Source: Front Immunol. 2026 Jul 9;17:1802168. doi: 10.3389/fimmu.2026.1802168 (PMC13391584; doi:10.3389/fimmu.2026.1802168)
Supplement: Supplementary file 1 [file Table1.docx]

***Supplementary Material***

# Supplementary Figures and Tables

## Supplementary Tables

| **Donor ID** | **1,25(OH)₂ vitamin D (pg/mL)** |
| --- | --- |
| Donor 1 | 41.4 |
| Donor 2 | 34.0 |
| Donor 3 | 67.2 |
| Donor 4 | 59.7 |
| Donor 5 | 47.9 |
| Donor 6 | 55.8 |
| Donor 7 | 52.4 |
| Donor 8 | 48.1 |
| Donor 9 | 44.5 |
| Donor 10 | 52.5 |

**Supplementary Table 1:** Plasma concentrations of 1,25(OH)₂ vitamin D in healthy donors (n = 10).

| **Gene** | **Primer Name** | **Sequence (5’ to 3’)** |
| --- | --- | --- |
| *β-Actin* | β-Actin-F | ACAGAGCCTCGCCTTTGC |
|  | β-Actin-R | CCACCATCACGCCCTGG |
| *PDCD1* | PDCD1-F | CGTGGCCTATCCACTCCTCA |
|  | PDCD1-R | ATCCCTTGTCCCAGCCACTC |
| *CTLA4* | CTLA4-F | CTCAGCTGAACCTGGCTACC |
|  | CTLA4-R | TGGGCACGGTTCTGGATCAAT |
| *HAVCR2* | HAVCR2-F | TGGTCATCAAACCAGCCAAG |
|  | HAVCR2-R | TGGCCAATCTAGAGTCCCGT |
| *TIGIT* | TIGIT-F | CTTCAAGGATCGAGTGGCCC |
|  | TIGIT-R | TGGATTCTGAGGGCTTTCTTCTTT |
| *IFN-γ* | IFN-γ-F | TTCAGCTCTGCATCGTTTTG |
|  | IFN-γ-R | TCTTTTGGATGCTCTGGTCA |

**Supplementary Table 2:** List of primers used for quantitative PCR (qPCR) analysis.

| **Assay** | **Marker** | **Fluorochrome** | **Clone** | **Catalog number** | **Supplier** |
| --- | --- | --- | --- | --- | --- |
| IC markers | CD3 | BV480 | UCHT1 | 566105 | BD Horizon |
| IC markers | CD8 | BV605 | SK1 | 564116 | BD Horizon |
| IC markers | CD4 | APC | SK3 | 566915 | BD Pharmingen |
| IC markers | CTLA-4 (CD152) | BV786 | BNI3 | 563931 | BD Horizon |
| IC markers | PD-1 (CD279) | PE-CF594 | EH12.1 | 565024 | BD Horizon |
| IC markers | TIM-3 (CD366) | BB515 | 7D3 | 565569 | BD Horizon |
| IC markers | TIGIT | BV421 | 741182 | 747844 | BD OptiBuild |
| AIM assay | CD3 | BV480 | UCHT1 | 566105 | BD Horizon |
| AIM assay | CD8 | BV605 | SK1 | 564116 | BD Horizon |
| AIM assay | CD4 | APC | SK3 | 566915 | BD Pharmingen |
| AIM assay | CD69 | APC-R700 | FN50 | 565154 | BD Horizon |
| AIM assay | CD137 | BV711 | 4B4-1 | 740798 | BD OptiBuild |
| CD8⁺ T cell phenotyping | CD3 | BV480 | UCHT1 | 566105 | BD Horizon |
| CD8⁺ T cell phenotyping | CD8 | BV605 | SK1 | 564116 | BD Horizon |
| CD8⁺ T cell phenotyping | CCR7 | BB700 | 3D12 | 566437 | BD Horizon |
| CD8⁺ T cell phenotyping | CD45RA | BB515 | HI100 | 566438 | BD Horizon |

**Supplementary Table 3:** Antibodies used for flow cytometry analyses.

| **Donor ID** | **CTR**  **(SFU/10⁶ PBMCs)** | **10 nM**  **(SFU/10⁶ PBMCs)** | **100 nM**  **(SFU/10⁶ PBMCs)** |
| --- | --- | --- | --- |
| Donor 1 | 1305 | 1535 | 1140 |
| Donor 2 | 3405 | 2605 | 3340 |
| Donor 3 | 580 | 660 | 165 |
| Donor 4 | 2980 | 3350 | 2760 |
| Donor 5 | 225 | -85 | 5 |
| Donor 6 | 1825 | 2335 | 2110 |
| Donor 7 | 610 | 715 | 870 |
| Donor 8 | 2940 | 3215 | 1060 |
| Donor 9 | 1470 | 1735 | 1255 |
| Donor 10 | 1515 | 1170 | 1075 |
| Donor 11 | 5425 | 3630 | 3725 |
| Donor 12 | 1370 | 125 | 100 |
| Donor 13 | 1310 | 430 | 110 |

**Supplementary Table 4:** Absolute IFN-γ ELISpot responses in PBMCs from 13 donors stimulated in the absence (CTR) or presence of 1,25(OH)₂ vitamin D (10 or 100 nM). Values represent background-subtracted IFN-γ SFU/10⁶ PBMCs, calculated as CMV-stimulated wells minus unstimulated wells, and are shown as the mean of two technical replicates for each donor and condition. Negative background subtracted values were retained as calculated for statistical analyses.

| **Cohort 1** | **1,25(OH)₂ vitamin D plasma levels** | **IFN-γ ELISpot** | **IFN-γ qPCR** | **Immune checkpoint qPCR** | **FACS** |
| --- | --- | --- | --- | --- | --- |
| Donor 1 | ✓ | ✓ | ✓ | ✓ | ✓ |
| Donor 2 | ✓ | ✓ | ✓ | NA | NA |
| Donor 3 | ✓ | ✓ | ✓ | NA | ✓ |
| Donor 4 | ✓ | ✓ | ✓ | ✓ | ✓ |
| Donor 5 | ✓ | ✓ | ✓ | ✓ | ✓ |
| Donor 6 | ✓ | ✓ | ✓ | ✓ | ✓ |
| Donor 7 | ✓ | ✓ | ✓ | NA | NA |
| Donor 8 | ✓ | ✓ | ✓ | NA | NA |
| Donor 9 | ✓ | ✓ | ✓ | ✓ | ✓ |
| Donor 10 | ✓ | ✓ | ✓ | NA | ✓ |
| Donor 11 | NA | ✓ | NA | NA | ✓ |
| Donor 12 | NA | ✓ | NA | NA | NA |
| Donor 13 | NA | ✓ | NA | NA | NA |

**Supplementary Table 5: Distribution of donor samples across experimental assays (cohort 1).** Check marks (✓) indicate donor samples included in each experimental analysis. All donors were included in IFN-γ ELISpot analyses (n = 13), whereas only donors with sufficient PBMC availability and/or adequate RNA after quality control were included in flow cytometry and qPCR analyses. NA = sample not available.

| **Cohort 2** | **CD107a degranulation** | **AIM analyses** | **Naïve/Effector phenotyping** | **FluoroSpot assay** |
| --- | --- | --- | --- | --- |
| Donor 1 | ✓ | ✓ | ✓ | ✓ |
| Donor 2 | ✓ | ✓ | ✓ | ✓ |
| Donor 3 | ✓ | ✓ | ✓ | ✓ |
| Donor 4 | ✓ | ✓ | ✓ | ✓ |
| Donor 5 | ✓ | ✓ | ✓ | ✓ |
| Donor 6 | ✓ | ✓ | ✓ | ✓ |
| Donor 7 | ✓ | ✓ | ✓ | ✓ |
| Donor 8 | ✓ | ✓ | ✓ | ✓ |

**Supplementary Table 6: Distribution of donor samples across experimental assays (cohort 2).** Check marks (✓) indicate donor samples included in each experimental analysis. All donors included CD107a-degranulation, AIM analyses, Naïve/Effector phenotyping and FluoroSpot Plus Assay.

| **Cytokine** | **CTR (mean ± SD)** | **100 nM VitD (mean ± SD)** | **Mean difference** | **95% CI** | **% change vs. CTR** | **p value** |
| --- | --- | --- | --- | --- | --- | --- |
| IL-4 | 1.000 ± 0.000 | 0.749 ± 0.481 | -0.251 | -0.653 to 0.151 | -25.1% | 0.148 |
| IL-5 | 1.000 ± 0.000 | 0.700 ± 0.448 | -0.300 | -0.674 to 0.074 | -30.0% | 0.250 |
| IL-22 | 1.000 ± 0.000 | 0.406 ± 0.430 | -0.595 | -0.954 to -0.235 | -59.5% | 0.015 |
| IL-10 | 1.000 ± 0.000 | 1.872 ± 2.294 | +0.872 | -1.046 to 2.790 | +87.2% | 0.742 |
| IL-17A | 1.000 ± 0.000 | 0.898 ± 0.689 | -0.102 | -0.678 to 0.474 | -10.2% | 0.546 |

**Supplementary Table 7: Quantitative analysis of cytokine secretion following treatment with 100 nM 1,25(OH)₂ vitamin D.** PBMCs obtained from 8 healthy donors were treated with 100 nM 1,25(OH)₂ vitamin D, and cytokine secretion was evaluated by FluoroSpot Plus assay following TCR stimulation with anti-CD3 and anti-CD28 antibodies. Data are presented as normalized Spot Forming Units (SFU; mean ± SD) relative to untreated controls (CTR = 1). Statistical analysis was performed using the Wilcoxon matched-pairs signed-rank test. Mean differences, 95% confidence intervals (95% CI), percentage changes versus CTR, and exact two-tailed p values are reported.

| **Subset** | **Comparison** | **CTR (mean ± SD)** | **VitD (mean ± SD)** | **Mean difference** | **95% CI** | **Adjusted p value** |
| --- | --- | --- | --- | --- | --- | --- |
| CCR7^+^/CD45RA^+^ | CTR vs 10 nM | 3.929 ± 2.305 | 3.863 ± 3.404 | -0.066 | -1.819 to 1.686 | 0.992 |
| CCR7^+^/CD45RA^+^ | CTR vs 100 nM | 3.929 ± 2.305 | 4.915 ± 2.794 | +0.986 | -0.933 to 2.906 | 0.324 |
| CCR7^+^/CD45RA^-^ | CTR vs 10 nM | 1.970 ± 1.429 | 0.998 ± 0.487 | -0.973 | -2.418 to 0.473 | 0.179 |
| CCR7^+^/CD45RA^-^ | CTR vs 100 nM | 1.970 ± 1.429 | 1.343 ± 0.700 | -0.628 | -2.011 to 0.756 | 0.399 |
| CCR7^-^/CD45RA^-^ | CTR vs 10 nM | 41.350 ± 11.250 | 39.130 ± 15.130 | -2.225 | -12.140 to 7.689 | 0.770 |
| CCR7^-^/CD45RA^-^ | CTR vs 100 nM | 41.350 ± 11.250 | 39.380 ± 18.780 | -1.971 | -17.520 to 13.580 | 0.916 |
| CCR7^-^/CD45RA^+^ | CTR vs 10 nM | 52.760 ± 9.374 | 56.010 ± 13.030 | +3.250 | -5.579 to 12.080 | 0.526 |
| CCR7^-^/CD45RA^+^ | CTR vs 100 nM | 52.760 ± 9.374 | 54.350 ± 15.950 | +1.587 | -12.380 to 15.560 | 0.932 |

**Supplementary Table 8: Distribution of CD8⁺ T cell subset following treatment with 1,25(OH)₂ vitamin D.** PBMCs obtained from 8 healthy donors were treated with 10 nM or 100 nM 1,25(OH)₂ vitamin D and analyzed by flow cytometry to quantify naïve (CCR7⁺/CD45RA⁺), central memory (CCR7⁺/CD45RA⁻), effector memory (CCR7⁻/CD45RA⁻), and terminal effector memory/TEMRA (CCR7⁻/CD45RA⁺) CD8⁺ T-cell subsets. Data are presented as mean ± SD. Mean differences are reported as treated conditions minus untreated control (CTR). Statistical analysis was performed using repeated-measures one-way ANOVA with Geisser–Greenhouse correction followed by Dunnett’s multiple comparisons test.

## Supplementary Material and Methods

## FluoroSpot Plus assay

A FluoroSpot assay was performed according to the manufacturer’s instructions using FluoroSpot Plus kits (Mabtech, Sweden). Briefly, 2×10⁵ cells/well were seeded in 96-well plates pre-coated with capture monoclonal antibodies and incubated at 37°C in a humidified 5% CO₂ atmosphere in the presence of the appropriate stimuli. Cells were stimulated with anti-CD3 and anti-CD28 antibodies to induce TCR-mediated activation. After 36 h, cells were removed and plates were washed 5 times with PBS. Plates were then incubated for 2 h at room temperature with the corresponding detection antibody cocktail. After 5 washing steps, plates were incubated for 1 h at room temperature with the corresponding fluorophore-conjugated reagents. Subsequently, FluoroSpot enhancer solution was added according to the manufacturer’s instructions. Spots were detected and counted using the appropriate fluorescence channels on the IRIS 2 FluoroSpot/ELISpot reader (Mabtech, Sweden), according to the manufacturer’s settings. Data were reported as Spot Forming Units per 10⁶ PBMCs (SFU/10⁶ PBMCs). For Interleukin-4 (IL-4) and Interleukin-5 (IL-5) detection, FluoroSpot Plus kit Cat. No. FSP-011608-2 was used. Plates were pre-coated with capture monoclonal antibodies against IL-4 (clone IL4-I) and IL-5 (clone TRFK5). Detection was performed using anti-IL-4-biotin and anti-IL-5-WASP antibodies, followed by SA-550 and anti-WASP-640 fluorophore conjugates. For Interleukin-22 (IL-22), Interleukin-10 (IL-10), and Interleukin-17A (IL-17A) detection, FluoroSpot Plus kit Cat. No. FSP-18010703-2 was used. Plates were pre-coated with capture monoclonal antibodies against IL-17A (clone MT12A3), IL-22 (clone 9D7), and IL-10 (clone MT44.6). Detection was performed using anti-IL-22-DIG, anti-IL-10-biotin, and anti-IL-17A-WASP antibodies, followed by anti-DIG, SA-550, and anti-WASP fluorophore-conjugated reagent, respectively.

## Statistical analysis

Statistical analyses were performed with GraphPad PRISM software 8 (GraphPad Software, La Jolla, CA, USA) or R software (version 4.2.3). Analyses were conducted using donor-matched data, with each donor serving as its own control across experimental conditions (CTR, 10 nM and 100 nM 1,25(OH)₂ vitamin D). For parametric paired datasets, differences among groups were assessed using repeated-measures one-way ANOVA with Geisser-Greenhouse correction, followed by Tukey’s or Dunnett’s multiple-comparisons post hoc test, depending on the experimental design and comparisons performed. Tukey’s post hoc test was used for all-pairs comparisons, whereas Dunnett’s post hoc test was applied when treatment groups were compared exclusively against untreated controls. Each checkpoint marker was analyzed independently, and multiple-comparison correction was applied within each analysis. For selected non-parametric paired datasets, statistical significance was assessed using the Wilcoxon matched-pairs signed-rank test. For selected analyses, data were normalized to the corresponding untreated control for each donor (CTR=1), to reduce inter-donor variability and facilitate comparison of relative treatment-induced changes across experiments. For datasets presented as normalized to CTR=1, statistical analyses were performed on normalized donor-level values. In contrast, for datasets presented as percentage values, statistical analyses were performed on raw donor-level data. Data are presented as mean ± SD unless otherwise specified, and individual donor data points are shown whenever possible. P values < 0.05 were considered statistically significant.

## Supplementary Figures


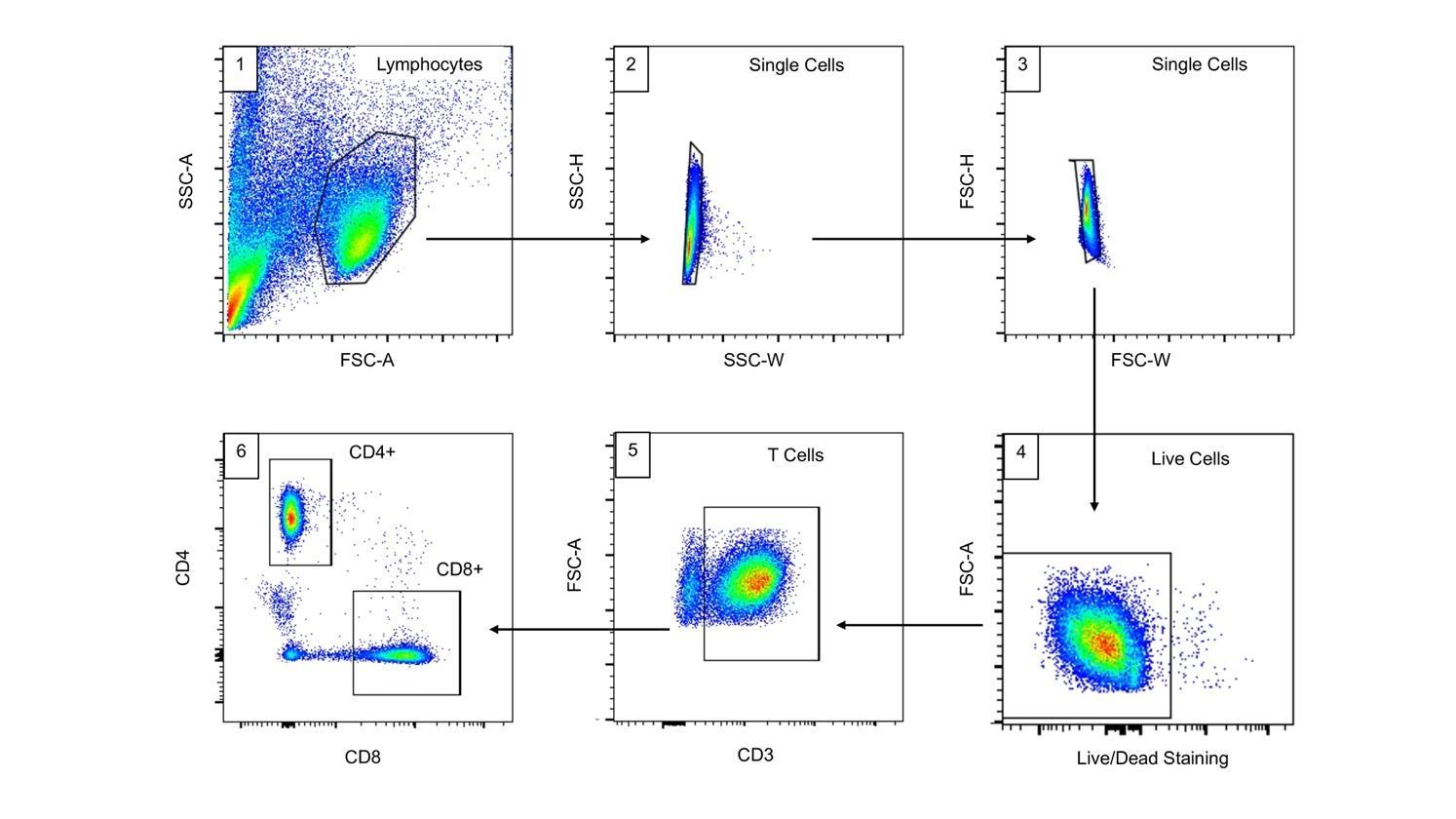
**Supplementary Figure 1:** **Flow cytometry gating strategy.** Example flow cytometry gating strategy for the determination of changes in the fluorescence intensity of immune checkpoint molecules in CD4^+^ or CD8^+^ T cells. Briefly, mononuclear cells were gated out of all events (1.) followed by subsequent singlet (2 and 3.) and live cells gating (4.). T cell population was then obtained using the CD3 marker (5.). The CD4 and CD8 markers were further used to define subpopulations within the CD3^+^ T cells (6.).

**
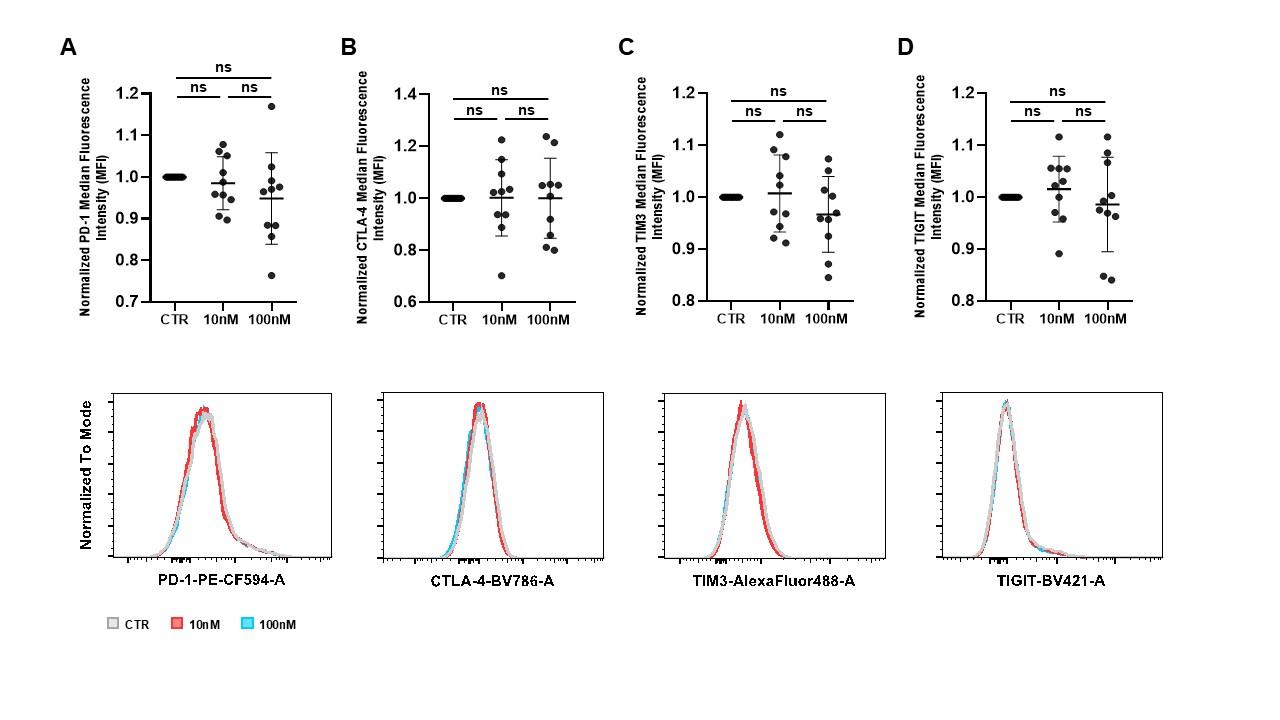
Supplementary Figure 2: Immune checkpoint markers in CD4^+^ T cells are not modulated upon 1,25(OH)₂ vitamin D treatment.** Surface expression of PD-1 **(A)**, CTLA-4 **(B)**, TIM-3 **(C)**, and TIGIT **(D)** on CD4^+^ T cells (n=10) was assessed by flow cytometry after stimulation of PBMCs in the absence (CTR) or presence of 1,25(OH)₂ vitamin D (10 or 100 nM). Upper panels show normalized median fluorescence intensity (MFI) values relative to untreated controls (CTR=1). Lower panels show representative histograms for each analyzed marker. Each dot represents an individual donor, and bars indicate mean ± SD. Statistical analysis was performed using repeated-measures one-way ANOVA with Geisser–Greenhouse correction followed by Tukey’s post hoc test.


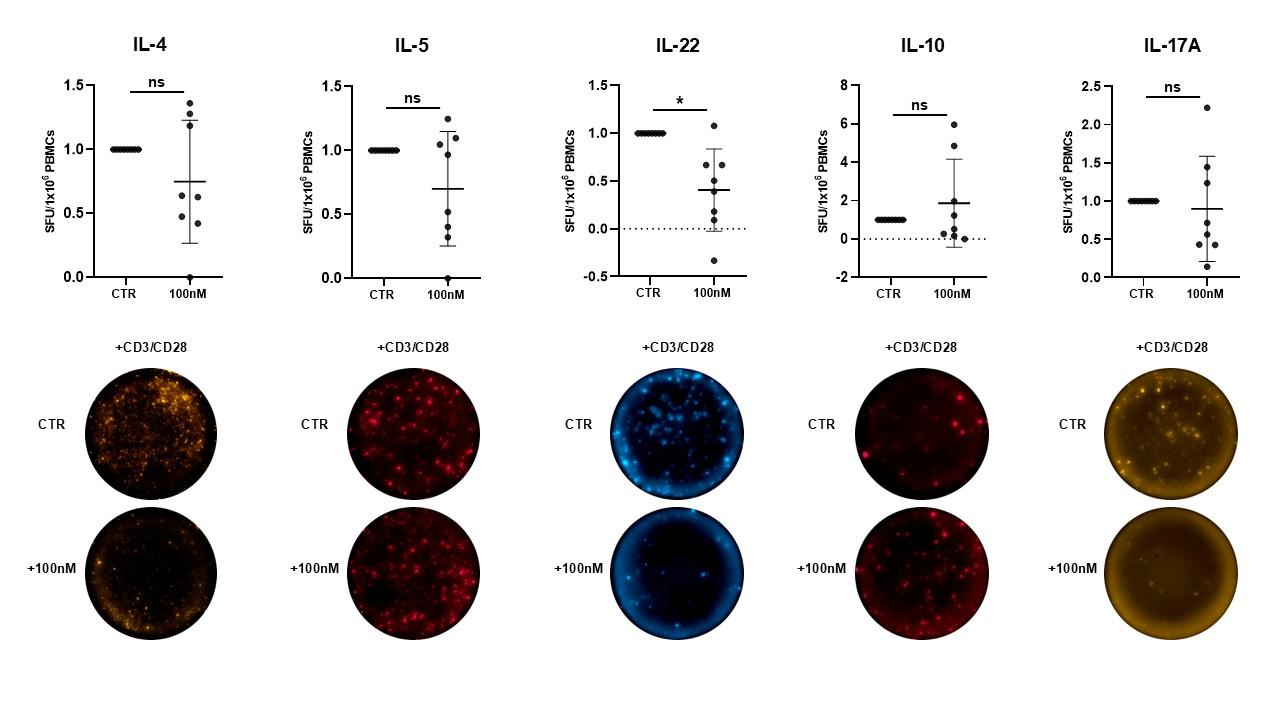
**Supplementary Figure 3: Effect of 1,25(OH)₂ vitamin D on CD4**^+^ **T cell-mediated cytokine production.** Plot showing the quantification of IL-4, IL-5, IL-22, IL-10, and IL-17A secretion measured by FluoroSpot assay following TCR stimulation with anti-CD3 and anti-CD28 antibodies. PBMCs obtained from 8 healthy donors were treated with 100 nM 1,25(OH)₂ vitamin D and cytokine secretion was normalized to untreated control (CTR=1). Data are presented as individual donors with normalized Spot Forming Units (SFU)/10⁶ PBMCs shown as mean ± SD. Representative FluoroSpot images for each cytokine are shown below the corresponding graphs. Statistical analysis was performed using the Wilcoxon matched-pairs signed-rank test.
